# The global research of artificial intelligence in lung cancer: a 20-year bibliometric analysis

**DOI:** 10.3389/fonc.2024.1346010

**Published:** 2024-02-02

**Authors:** Ruikang Zhong, Tangke Gao, Jinghua Li, Zexing Li, Xue Tian, Chi Zhang, Ximing Lin, Yuehui Wang, Lei Gao, Kaiwen Hu

**Affiliations:** ^1^ Beijing University of Chinese Medicine, Beijing, China; ^2^ Guang’an Men Hospital, China Academy of Chinese Medical Sciences, Beijing, China; ^3^ Dongfang Hospital, Beijing University of Chinese Medicine, Beijing, China

**Keywords:** artificial intelligence, lung cancer, bibliometrics, VOSviewer, Citespace, visualization

## Abstract

**Background:**

Lung cancer (LC) is the second-highest incidence and the first-highest mortality cancer worldwide. Early screening and precise treatment of LC have been the research hotspots in this field. Artificial intelligence (AI) technology has advantages in many aspects of LC and widely used such as LC early diagnosis, LC differential classification, treatment and prognosis prediction.

**Objective:**

This study aims to analyze and visualize the research history, current status, current hotspots, and development trends of artificial intelligence in the field of lung cancer using bibliometric methods, and predict future research directions and cutting-edge hotspots.

**Results:**

A total of 2931 articles published between 2003 and 2023 were included, contributed by 15,848 authors from 92 countries/regions. Among them, China (40%) with 1173 papers,USA (24.80%) with 727 papers and the India(10.2%) with 299 papers have made outstanding contributions in this field, accounting for 75% of the total publications. The primary research institutions were Shanghai Jiaotong University(n=66),Chinese Academy of Sciences (n=63) and Harvard Medical School (n=52).Professor Qian Wei(n=20) from Northeastern University in China were ranked first in the top 10 authors while Armato SG(n=458 citations) was the most co-cited authors. *Frontiers in Oncology*(121 publications; IF 2022,4.7; Q2) was the most published journal. while *Radiology* (3003 citations; IF 2022, 19.7; Q1) was the most co-cited journal. different countries and institutions should further strengthen cooperation between each other. The most common keywords were lung cancer, classification, cancer, machine learning and deep learning. Meanwhile, The most cited papers was Nicolas Coudray et al.2018.NAT MED(1196 Total Citations).

**Conclusions:**

Research related to AI in lung cancer has significant application prospects, and the number of scholars dedicated to AI-related research on lung cancer is continually growing. It is foreseeable that non-invasive diagnosis and precise minimally invasive treatment through deep learning and machine learning will remain a central focus in the future. Simultaneously, there is a need to enhance collaboration not only among various countries and institutions but also between high-quality medical and industrial entities.

## 1 Introduction

Lung cancer stands as the leading cause of cancer-related deaths globally and ranks as the second most commonly diagnosed cancer. The average 5-year survival rate is only 15% (1).With concerted efforts to advance CT early screening and update treatment methods, the latest epidemiological report on lung cancer in the United States indicates a continuous decline in the incidence rate and mortality of lung cancer (2).Consequently, the development of earlier and more accurate diagnosis, along with more precise and personalized treatment, holds significant importance for the prevention and prognosis of lung cancer.

Artificial Intelligence is a discipline that mainly studies the application of computers to simulate human intelligent behavior, involving various disciplines such as computation, mathematics, biology, etc (3).With the arrival of the big data era and the updates and progress of computer equipment and algorithm technology, artificial intelligence has been widely applied in multiple fields, gradually penetrating into all aspects of our lives. In the 1950s, the medical field had already noticed the potential of AI and began to attempt to apply AI to auxiliary diagnosis of diseases (4). In recent years, AI has gradually been widely applied in various aspects of healthcare with its powerful algorithms and learning capabilities, including disease diagnosis, prognosis prediction, drug research, genomics data analysis, etc., bringing new methods such as imaging omics, pathomics, genomics, etc. to the medical field (5). A mount of AI technologies such as machine learning (ML) and deep learning (DL) have been used for auxiliary diagnosis and prognosis prediction of lung cancer and achieved good predictive performance (6–9).More and more scholars are beginning to explore the application and implementation of AI in the field of lung cancer, and the number of research studies in this area is also growing exponentially. This makes it increasingly difficult for most researchers to keep up with the latest research findings, stay informed about research trends, and anticipate future developments.

Bibliometric analysis is an information visualization method that involves summarizing all literature globally in a specific field. It utilizes mathematical and statistical methods to quantitatively analyze bibliographic data and measurement characteristics. This process aims to comprehend the knowledge structure of a particular field and identify research frontiers or hotspots, representing a form of information visualization. Due to the rigorous and objective nature of bibliometric analysis, scholars in various fields commonly employ this method to conduct research in their respective domains (10). However, as of now, there are no bibliometric studies related to AI in lung cancer. Therefore, we aim to conduct a quantitative and qualitative analysis, along with visualization, of the research progress and current status in the field of artificial intelligence applied to lung cancer by collecting relevant literature from databases over the past 20 years. This endeavor is intended to provide insights into potential future research trends, aiding scholars in this field to develop a more systematic understanding of research priorities and future directions.

## 2 Methods

### 2.1 Data source and search strategies

Two independent authors conducted a relevant publications search on the Web of Science (http://webofscience.com;ThomsonReuters, Toronto, Canada). The data were collected from the Web of Science Core Collection (WoSCC) database. There are three reasons for choosing the WoSCC database: it covers a wide range of publication from different fields, it is considered one of the most influential databases and it is commonly used in bibliometric analysis. Eligible publications included those published between the 1st of January 2003 and the 31st of July 2023. The search was limited to the publications indexed in Science Citation Index Expanded (SCI-EXPANDED) and Social Sciences Citation Index (SSCI).

**Figure 1:**
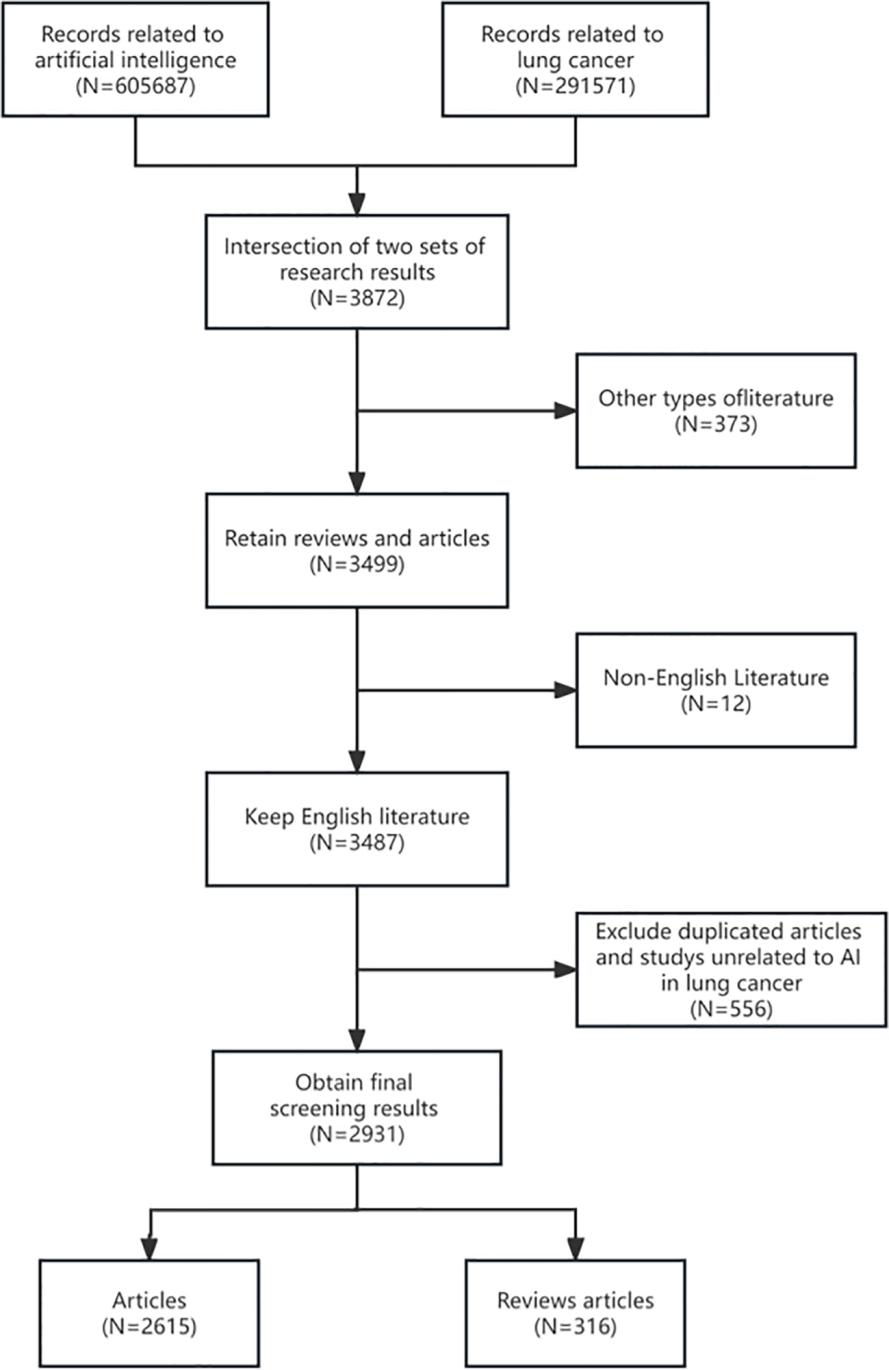
Flowchart of the publications selection in the study.

“Artificial intelligence” and “Lung cancer” were used as search terms, with their relevant synonyms or abbreviations. Then compared their respective findings to ensure the integrity and accuracy of search results. The search query was TS= (“artificial intelligence” OR “deep learning” OR “artificial Neural Network” OR “computer vision” OR “machine learning” OR “Knowledge graph” OR “neural network” OR “computational intelligence” OR “Data mining” OR “Supervised Learning” OR “Unsupervised Learning” OR “Convolutional Neural Network” OR “transfer learning” OR “Reinforcement Learning”) AND TS= (“lung cancer” OR “lung tumor” OR “pulmonary ground-glass” OR “lung malignancy” OR “lung carcinoma” OR “lung metastasis” OR “lung metastatic” OR “pulmonary metastatic” OR “pulmonary metastasis”). The detailed search strategy is shown in **Figure 1**.

According to our research field, ethical approval is not required for the current study as the data were retrospectively downloaded from databases. It is worth mentioning that, in our search strategy, articles related to lung cancer were included not only for their direct relevance but also due to their association with breast cancer, colon cancer, and thoracic tumors. These articles are generally applied in differential diagnosis among different types of cancers and in distinguishing between metastatic and primary cancers. Additionally, a small proportion of articles were included based on mentions of lung cancer in the introductory sections of epidemiological descriptions. Therefore, such articles need to be manually excluded with precision. Simultaneously, exclusions were made for articles related to environmental and humanities aspects of lung cancer risk factors, which might have been included due to keyword searches.

### 2.2 Analysis tools

Using two tools for bibliometric analysis, namely VOSviewer and CiteSpace. VOSviewer is a widely utilized bibliometric tool that specializes in visualizing bibliometric networks (11). It can group closely related nodes into multiple clusters, with nodes of the same color indicating a higher level of correlation. Additionally, VOSviewer supports two visualization maps: overlay visualization and density visualization. This study primarily employs VOSviewer for co-authorship analysis among countries, institutions, and authors, as well as for analyzing co-citation of references.

CiteSpace is a free Java application that focuses on dynamic visualization, reflecting the evolution of bibliometric networks over time (12, 13). In this research, it is used for high-frequency keyword clustering, timeline visualization of keywords and highly cited references, and identification of the most influential references and keywords during specific periods. Furthermore, a generalized additive model based on the R and mgcv package is utilized to statistically analyze publication trends and citation counts. An online bibliometric platform (https://bibliometric.com/) is employed to visually represent international collaboration among countries.

### 2.3 Field analyses

Statistical analysis was conducted on the following fields: title, keywords, authors, author affiliations, author countries or regions, journal names, document names, total citation frequency, and annual average citation frequency. An investigation was carried out on the annual publication volume to observe the development of the discipline. A list was compiled of active countries, institutions, authors, and journals to identify influential scholars and organizations. To some extent, the quantity of citations constitutes the significance of research; hence, statistical analysis was performed. Based on the citation count, authors, author countries or regions, author affiliations, journals, and papers were ranked to determine their importance in the field. The H-index measures the productivity and citation impact at the author level (14). In comparison to other quantitative metrics, it provides a more comprehensive assessment of scholars’ work (15).

## 3 Results

### 3.1 Global trends of publication outputs and citations

**Figure 2:**
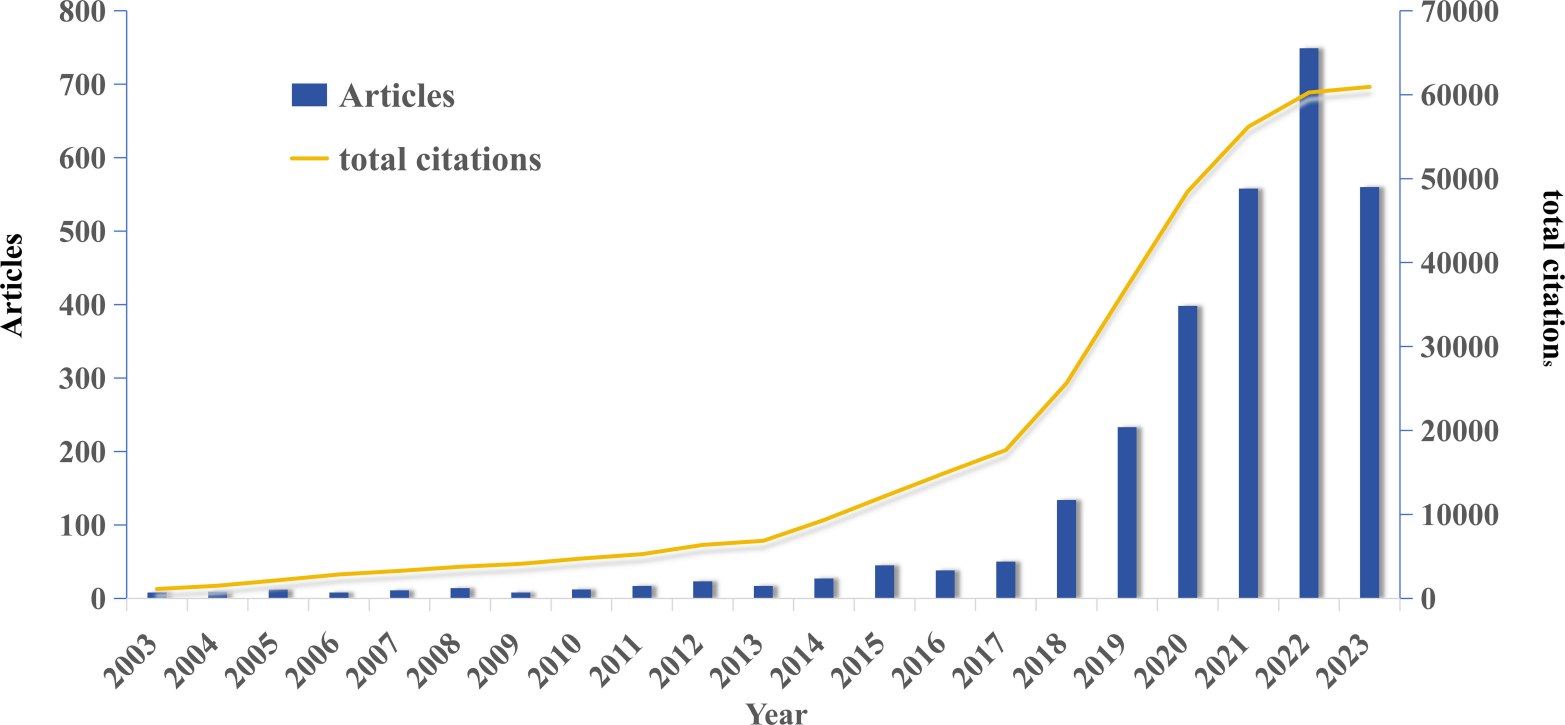
Global trend of publications and total citations on AI-based lung cancer research over the past 20 years.

This study retrieved a total of 2931 publications, including 2615 original articles and 316 reviews. As shown in **Figure 2**, the publication volume was relatively low before 2017, with an annual average growth rate of 18.86%. However, the number of publications has rapidly increased since then, with an annual average growth rate of 70.39%. In fact, 89.7% of the literature (2632 out of 2931) has been published in the last 6 years (2018-2023). This might be attributed to the rapid development and progress of machine learning and deep learning in both computing resources and algorithms in recent years, leading to an increasing number of publications during this period. It is anticipated that the number of publications in 2023 will eventually reach 1020. The total citation count follows a similar trend, also experiencing rapid growth after 2017.

### 3.2 Contributions of countries and regionals

**Figure 3:**
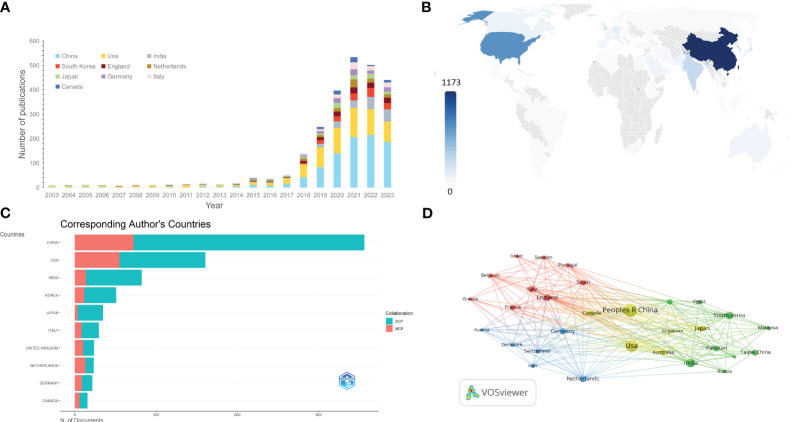
**(A)** The changing trend of the annual publication quantity in the top 10 countries/regions over the past 23 years. **(B)** Geographic distribution map based on the total publications of different countries/regions. **(C)** Top 10 most productive countries chart, divided by single country publications (SCPs) and multiple country publications (MCPs). **(D)** The countries/regions citation overlay visualization map generated by using VOS viewer.

A total of 79 countries/regions have contributed to AI-based lung cancer research. In **Table 1**, the top 10 countries are listed by publication volume, with China, the United States, India, South Korea, and England leading in publication outputs. Together, China and the United States contribute to over 50% of the total publications. The Netherlands, Canada, Germany, the United States, and England have the highest average citation counts. **Figure 3A** illustrates the changes in publication output for the top 10 countries from 2003 to 2023, with the United States leading in publication volume until 2019 when it was surpassed by China, ranking second. In **Figure 3B**, the world map shows that publications in this field predominantly originate from North America and East Asian countries.

**Table 1:** Top 10 productive countries/regions related to AI on LC.

Rank	Country	Publication	Percentage	Citation	Average Citation
1	China	940	32.07	18418	19.59
2	USA	649	22.14	27116	41.78
3	India	188	6.41	3965	21.09
4	South Korea	134	4.57	2761	20.60
5	England	116	3.96	4739	40.85
6	The Netherlands	112	3.82	7600	67.86
7	Italy	106	3.62	3032	28.60
8	Japan	106	3.62	2762	26.06
9	Germany	97	3.31	4083	42.09
10	Canada	75	2.56	3414	45.52

From the statistics of the single country publications(SCP) and multiple country publications(MCP), it can be seen that among the top 10 countries, most research consists of single-country studies, with the Netherlands being the only exception, having collaborative research exceeding single-country studies (**Figure 3C**). Utilizing a normalized Louvain clustering algorithm based on associations, a cooperative network analysis was conducted for the top 30 most productive countries. Isolated nodes were removed, considering a minimum edge weight of 1. Four major collaboration clusters were identified: the first cluster involving China and the United States, representing the most productive participants; the second cluster focused on the Asian region (excluding Russia), primarily involving India and South Korea; the third and fourth clusters concentrated in the European region, one led by the Netherlands and Germany, and the other involving England, Italy, Spain, France, and other countries (**Figure 3D**).

### 3.3 Analysis of top institutions and funding agencies

**Figure 4:**
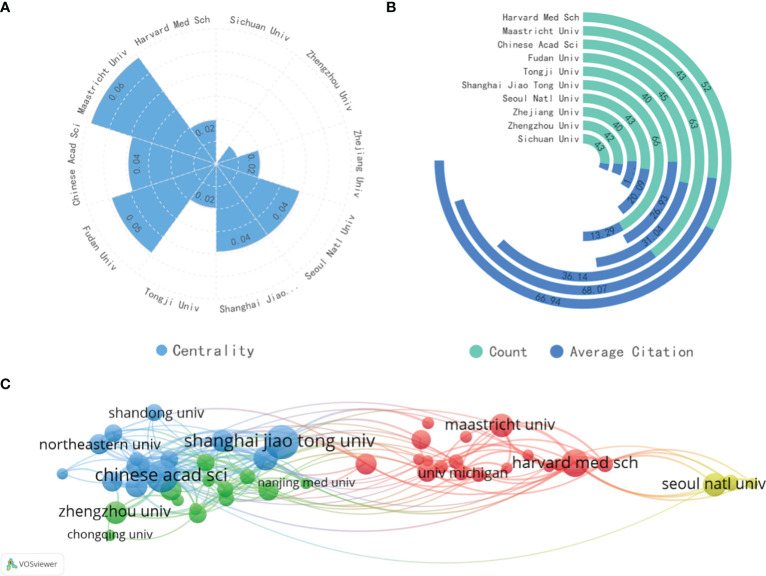
**(A)** The centrality of the top 10 most productive organizations. **(B)** The total publications and total citations of the top 10 institutions. **(C)** The visualization map of institutions co-authorship analysis generated by VOSviewer.

A total of 4038 institutions were included in this study. Among them, Shanghai Jiao Tong University in China has the highest publication output (n=66), followed by the Chinese Academy of Sciences (n=63) and Harvard Medical School (n=52). As evident from **Table 2** and **Figures 4A, B**, among the top 10 institutions with the highest publication output, Chinese institutions account for 70%, indicating active research in this field from China. Harvard Medical School and Maastricht University have the highest total citation counts and average citation counts, holding the top two positions. They exhibit notable centrality, suggesting significant influence in the research related to AI in lung cancer. This underscores the substantial impact and authority of these two institutions on scholars engaged in AI in LC research.

**Table 2:** Top 10 productive organization related to AI on LC.

Rank	Organization	Count	Total Citation	Average Citation	Centrality	Country
1	shanghai jiao tong univ	66	877	13.29	0.04	China
2	chinese acad sci	63	2277	36.14	0.04	China
3	harvard med sch	52	3481	66.94	0.02	USA
4	fudan univ	45	1397	31.04	0.05	China
5	maastricht univ	43	2927	68.07	0.06	The Netherlands
6	seoul natl univ	43	864	20.09	0.04	South Korea
7	sichuan univ	43	282	6.56	0	China
8	zhengzhou univ	42	303	7.21	0.01	China
9	tongji univ	40	1077	26.93	0.02	China
10	zhejiang univ	40	542	13.55	0.02	China

The top 50 institutions by publication volume were imported into VOSviewer to generate a clustering diagram, as shown in **Figure 4C**. The diagram reveals three main clusters. The blue and green clusters, primarily consisting of Chinese institutions, suggest a close collaboration among these Chinese organizations. The red cluster, led by Harvard Medical School and Maastricht University, encompasses institutions mainly from North America and Europe, indicating that influential institutions can foster international cooperation. The yellow cluster, centered around Seoul National University, Chung-Ang University, and other South Korean institutions, forms a relatively isolated group with limited collaboration with the other two clusters.

Funding agencies played a key role in the conduct of research and the publication of articles. In the sight of that, **Table 3** summarized the top 10 funding agencies by publications. From the results, there were a total of 5 funding agencies from China and 3 from the United States, of which the National Natural Science Foundation of China, National Institutes of Health and the National Key Research and Development Program of China occupied the top three in this field. This result clearly demonstrated that the United States’ and China’s leading position as well as China’s rapid development in this field were closely related to their strong economic foundation and support.

**Table 3:** The top most active funding agencies in AI-based tumor pathology research.

Rank	Awards	Count	Centrality	Country
1	National Natural Science Foundation of China	368	0.47	China
2	National Institutes of Health	80	0.17	USA
3	National Key Research and Development Program of China	41	0.03	China
4	National Key R&D Program of China	39	0.03	China
5	Fundamental Research Funds for the Central Universities	29	0.03	China
6	National Research Foundation of Korea	27	0.06	South Korea
7	Grants-in-Aid for Scientific Research	25	0.04	Japan
8	National Cancer Institute	23	0.08	USA
9	NCI NIH HHS	19	0	USA
10	Beijing Natural Science Foundation	16	0.01	China

### 34 Analysis of the active authors and co−cited authors

**Figure 5:**
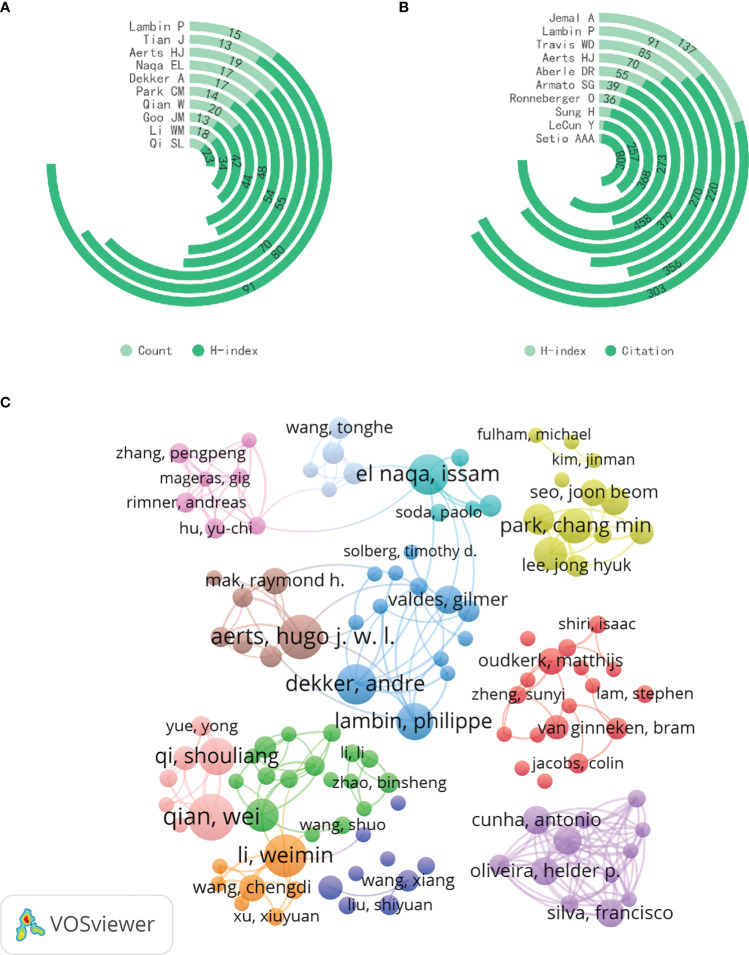
**(A)** The total publications and H-index of the top 10 most productive authors. **(B)** The total citations and H-index of the top 10 10 co-cited authors. **(C)** The visualization map of author co-authorship analysis generated by VOSviewer.

In total, 15,838 different authors and 57,100 co-cited authors were included in the analyzed literature, with an average of 7.89 authors per paper. The top 10 authors by publication volume and co-citation count were identified and visualized to determine key contributors in the field (**Table 4**, **Figures 5A, B**). Professor Wei Qian (H-index=44) has the highest publication volume and is an honorary professor at Northeastern University in China and a tenured professor at the University of Texas. His research focuses on computer-aided cancer diagnosis (16), medical big data analysis (17), and computer-aided analysis of cancer treatment plans (18, 19). His significant contributions lie in the efficient and robust computer-aided analysis system simulation, modeling, design, and implementation based on medical images such as lung CT images, cell images, molecular images, and artificial intelligence technologies, including artificial neural networks, fuzzy logic, genetic algorithms, and evolutionary algorithms.

**Table 4:** The top 10 most productive authors and top 10 co-cited authors.

Rank	Author	Count	Centrality	H-index	Country	Co-cited Author	Citation	Centrality	H-index	Country
1	Qian W	20	0	44	China	Armato SG	458	0.39	39	USA
2	Aerts HJ	19	0.02	70	USA	Aberle DR	379	0.17	55	USA
3	Li WM	18	0.01	34	China	Sung H	368	0.01	25	USA
4	Dekker A	17	0.01	54	The Netherlands	Lambin P	356	0.15	91	The Netherlands
5	Naqa EL	17	0.01	55	USA	Setio AAA	308	0.12	12	The Netherlands
6	Lambin P	15	0.01	91	The Netherlands	Jemal A	303	0.16	137	USA
7	Qi SL	15	0	23	China	Ronneberger O	273	0.07	36	UK
8	Park CM	14	0	48	South Korea	Aerts HJ	270	0.08	70	USA
9	Goo JM	13	0	42	South Korea	LeCun Y	257	0.03	13	USA
10	Tian J	13	0.04	80	China	Travis WD	220	0.04	85	USA

Dr. Samuel Armato (H-index=39) from The University of Chicago Medicine Comprehensive Cancer Center is the most co-cited author, specializing in computer-aided diagnosis of chest imaging, including lung cancer screening and the diagnosis of malignant pleural mesothelioma. The LIDC/IDRI Lung Nodule Database (20), established under his leadership in 2011, is currently the largest and most widely used CT scan lung nodule database, making significant contributions to lung cancer early screening research. Notably, Hugo J.W.L.Aerts (H-index=70) from Harvard Medical School and Philippe Lambin (H-index=91) from Maastricht University are among the top ten authors in both publication volume and co-citation count in the AI in LC field.

**Figure 5C** is a co-authorship analysis visualization generated by VOSviewer. Although there is some international collaboration among researchers like Philippe Lambin, Hugo J.W.L.Aerts, and Issam EL Naqa (H-index=55), the research clusters are generally dispersed, with a concentration within the same country or institution, indicating limited international.

### 35 Analysis of top journals and co−cited journals

In this study, all publications related to the application of artificial intelligence in lung cancer are distributed across 723 academic journals and 16,938 co-cited journals. **Table 4** summarizes the top 20 journals and co-cited journals, incorporating publication volume, total citation count, Impact Factor (IF), and Journal Citation Reports (JCR) category to comprehensively assess the influence of journals. As shown in **Table 4**, except for *International Journal of Imaging Systems and Technology* all the top 20 journals are either Q1 or Q2, with 30% of journals and 70% of co-cited journals belonging to Q1. These journals predominantly focus on the intersection of the medical and computer engineering fields, with many specifically dedicated to interdisciplinary research between the two.

Publication volume reflects a journal’s attention and activity in the field, to some extent indicating the research frontiers and development trends in the domain. *Frontiers in Oncology*(121 publications) has the highest output in the application of AI in LC, followed by *Scientific Reports* (93 publications) and *Cancers* (88 publications). Co-citation frequency reflects whether a journal has had a significant impact on a research field, determining its influence. *Radiology* (cited 3003 times) has the highest co-citation frequency, followed by *Medical Physics* (cited 2969 times), *Scientific Reports* (cited 2563 times). As we can see, *Scientific Reports* ranks second among top journals and third among co-cited journals, indicating its significant impact on the application of AI in LC. Additionally, two highly co-cited sources, *Lecture Notes in Computer Science* (cited 1583 times, a classic work in computer science) and *IEEE Conference on Computer Vision and Pattern Recognition* (cited 1397 times, a top conference in computer vision), are excluded from the co-cited journal rankings as they are not journals but remain noteworthy.

### 3.6 Keyword analysis

**Figure 6:**
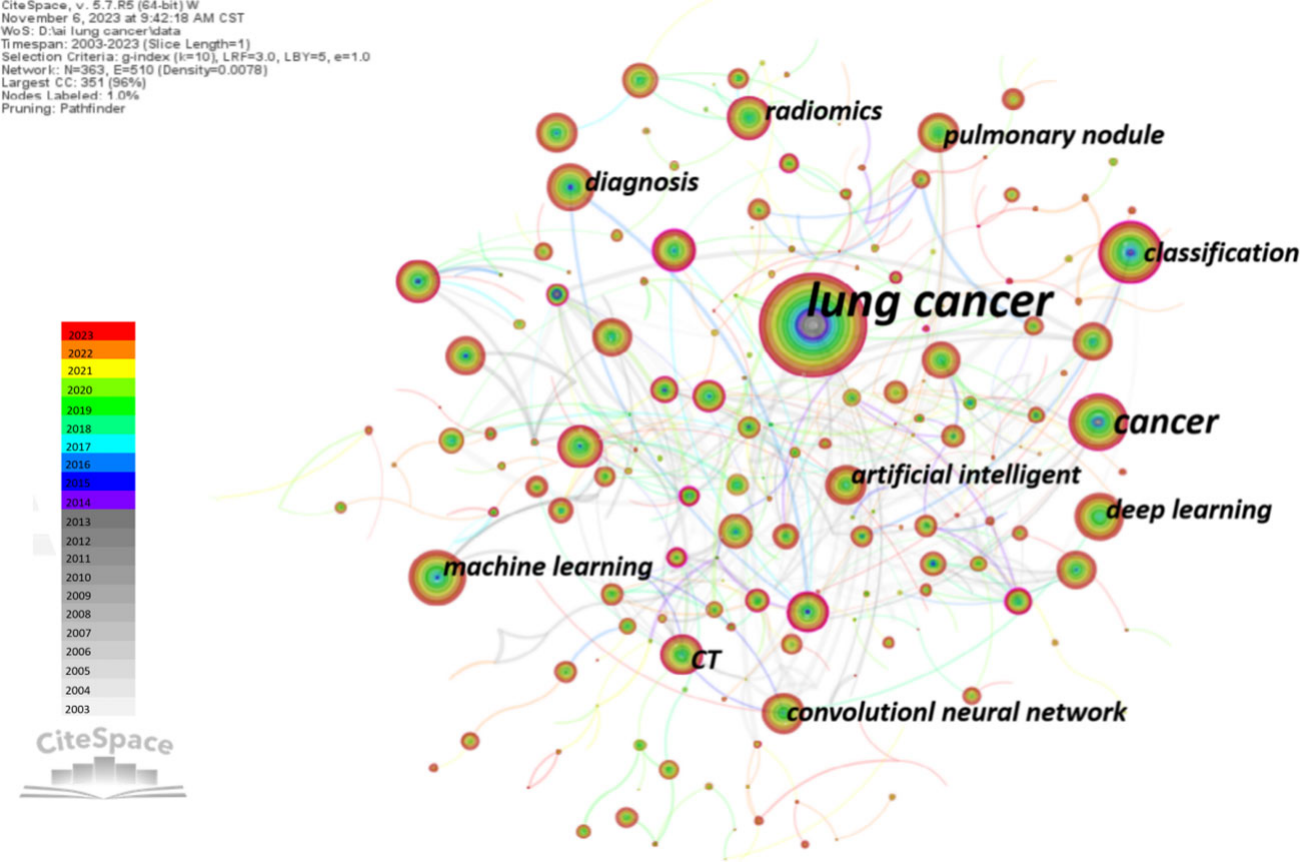
The overlay visualization map of author keywords co-occurrence analysis.

Keywords not only help observe the correlation between research topics but also contribute to understanding the current status and hotspots in a particular field. We extracted keywords from these documents for analysis. The total sum of keywords in 1,531 documents is 5,203, with 107 keywords appearing more than 20 times. Using CiteSpace software, we obtained a co-occurrence knowledge map of keywords (**Figure 6**). In the visualization map, larger nodes represent more frequent co-occurrences of keywords. Additionally, the thickness of the lines indicates the strength of co-occurrence between nodes; the thicker the line, the stronger the co-occurrence. Therefore, keywords with higher co-occurrence frequency and centrality are more important in the research field (21). As shown in **Figure 6**, the node for “lung cancer” is the largest, followed by “classification,” “cancer,” “machine learning,” and “deep learning.”

**Figure 7:**
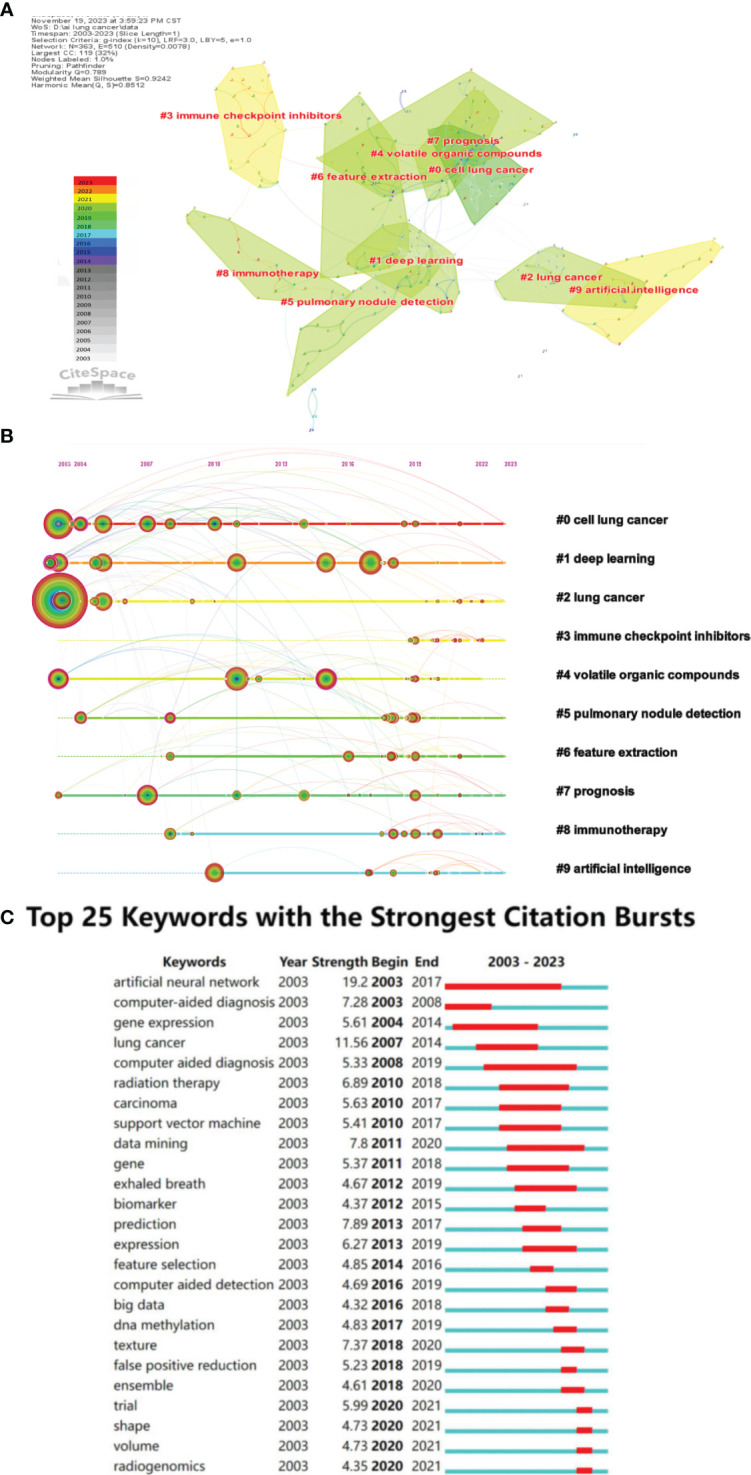
**(A)** The cluster view map of keyword. **(B)** The cluster timeline view map of keywords analysis. **(C)** Visualization map of top 25 keywords with the strongest citation bursts in AI-based lung cancer research.

Additionally, we utilized CiteSpace software to obtain the clustering function shown in **Figure 7A**. It is worth noting that modularity value (Q value) and average silhouette value (S value) are two important indicators for evaluating the significance of community structure. When Q > 0.3 and S > 0.7, the clustering is considered significant (22). In the network map, there are a total of 16 distinct clusters, and the Q value (0.789) and weighted average silhouette (0.9242) confirm the rationality of this network. From **Figure 7A**, it can be observed that “cell lung cancer” #0 and “deep learning” #1 are the largest clusters, followed by “lung cancer” #2, “immune checkpoint inhibitors” #3, and “volatile organic compounds” #4.

In order to further analyze the keywords related to the application of AI in LC, a Timeline View analysis was conducted. For temporal clustering, “Find Clusters” was clicked, then “LLR,” and finally “Timeline View” was selected in the Layout, with the results shown in **Figure 7B**. Examining the evolutionary speed of each cluster over time allows for a more in-depth exploration of the key research topics in the field from a micro perspective. In **Figure 7B**, there are a total of 16 clusters, numbered from 0 to 15. Additionally, the distance from left to right for each cluster represents the start and end times of each cluster, the size of the color-loaded points indicates the frequency of occurrence of the cluster’s label terms, and the color lines represent co-occurrence relationships between different cluster label terms. It can be observed that terms such as “lung cancer,” “deep learning,” “classification,” “volatile organic compounds,” and “prognosis” appeared early and have consistently been research hotspots. In recent years, research hotspots have focused on areas such as deep learning, immunotherapy, lung nodules, and imaging genomics. In Cluster 0, “gene” first appeared as a high-frequency term in lung cancer in 2010. People realized the gradually important role of genes in the diagnosis and treatment of lung cancer. With the rise of targeted and immunotherapy, the high-frequency term “gene mutation” appeared in 2019. With the development of artificial intelligence, “transfer learning” also emerged. In Cluster 1, on the timeline of deep learning, there has consistently been the emergence of high-frequency terms. Notably, in 2005, “lung nodules” began to appear as a high-frequency term, closely related to the high-frequency term “computer-aided diagnosis” in Cluster 7. Based on the keyword distribution, chest CT remains the primary means of AI for determining the malignancy of lung nodules. In Cluster 2, “neural networks” and “lung cancer” appeared around the same time. In the past five years, early diagnosis of lung cancer remains a high-frequency term. The appearance of “COVID-19” as a high-frequency term in 2020 suggests researchers are attempting to analyze the correlation between COVID-19 and lung cancer using artificial intelligence. In Cluster 3, high-frequency terms mainly appeared after 2018, with a relatively uniform distribution of keywords. This indicates that researchers gradually recognized the importance of immunotherapy in the prevention and treatment of lung cancer, and they are exploring it with the assistance of artificial intelligence. In Cluster 5, “automated detection” first appeared as a high-frequency term in 2003. In the past five years, with the development of CT and AI technologies, the imaging-assisted diagnosis of lung nodules has become increasingly sophisticated, primarily relying on CT. In Cluster 7’s timeline, “computer-aided detection” was first proposed around 2014 and has been a research hotspot since then. In Cluster 8, “immunotherapy” first appeared around 2008, along with “computer-aided diagnosis.” Afterward, no high-frequency terms appeared until 2017, when the development of imaging technology led to the high-frequency occurrence of “positron emission tomography” Around 2020, there is a significant increase in the application of artificial intelligence in immunotherapy for lung cancer. In Cluster 9, “artificial intelligence” first appeared as a keyword around 2010 and has been continuously under attention. In the past five years, keywords mainly focused on aspects such as “survival prediction”, “lung metastasis”, and “heterogeneity”.

Moreover, the burst detection algorithm developed by Kleinberg (23) is an effective analytical tool used to capture turning points in the popularity of keywords or citations during a specified period. **Figure 7C** displays the top 25 keywords with the strongest bursts. The blue line represents the time interval, and the red line represents the duration of the burst. The keyword with the highest burst intensity is “AI,” first appearing in 2003. After 2015, the duration of bursts for keywords gradually shortened.

### 3.7 Most cited papers and references

This study includes a total of 2,931 papers, with 118 papers having more than 100 citations. **Table 5** presents the top 10 papers ranked by citation count. The most cited paper (1196 citations) is the research on deep learning and pathology predicting NSCLC classification and mutations published by Nicolas Coudray and colleagues in 2018 (24) following that are Katherine A. Hoadley et al. and Diego Ardila et al. Except for Katherine A Hoadley et al, these ten papers primarily focus on the application of AI in medical imaging and histopathological images of lung cancer.

**Table 5:** The top 20 journals and co-cited journals.

Rank	Journal	Output	IF	JCR	Co-cited journal	citation	IF	JCR
1	Frontiers in Oncology	121	4.7	Q2	Radiology	3003	19.7	Q1
2	Scientific Reports	93	4.6	Q2	Medical Physics	2969	3.8	Q2
3	Cancers	88	5.2	Q1	Scientific Reports	2563	4.6	Q2
4	Ieee Access	75	3.9	Q2	Ieee Transactions on Medical Imaging	2013	10.6	Q1
5	Medical Physics	75	3.8	Q2	New England Journal of Medicine	1904	158.5	Q1
6	Computers in Biology And Medicine	50	7.7	Q1	plos one	1887	3.7	Q2
7	Diagnostics	48	3.6	Q2	Journal of Thoracic Oncology	1705	20.4	Q1
8	Physics in Medicine And Biology	43	3.5	Q2	International Journal of Radiation Oncology	1542	7	Q1
9	Applied Sciences-basel	37	2.7	Q2	Nature	1533	64.8	Q1
10	Plos One	36	3.7	Q2	Medical Image Analysis	1474	10.9	Q1
11	Biomedical Signal Processing And Control	35	5.1	Q2	Journal of Clinical Oncology	1424	45.3	Q1
12	European Radiology	31	5.9	Q1	Physics in Medicine And Biology	1416	3.5	Q2
13	Computer Methods And Programs in Biomedicine	30	6.1	Q1	European Radiology	1311	5.9	Q1
14	Journal of Digital Imaging	28	4.4	Q2	Clinical Cancer Research	1216	11.5	Q1
15	Sensors	28	3.9	Q2	Radiotherapy And Oncology	1215	5.7	Q1
16	Expert Systems with Applications	26	8.5	Q1	Lung Cancer	1161	5.3	Q1
17	Multimedia Tools And Applications	26	3.6	Q2	CA-A Cancer Journal for Clinicians	1128	254.7	Q1
18	Frontiers in Immunology	23	7.3	Q1	Frontiers in Oncology	1090	4.7	Q2
19	International Journal of Imaging Systems And Technology	22	3.3	Q3	Ieee Access	1064	3.9	Q2
20	Translational Lung Cancer Research	22	4.0	Q2	Cancer Research	1037	11.2	Q1

**Figure 8:**
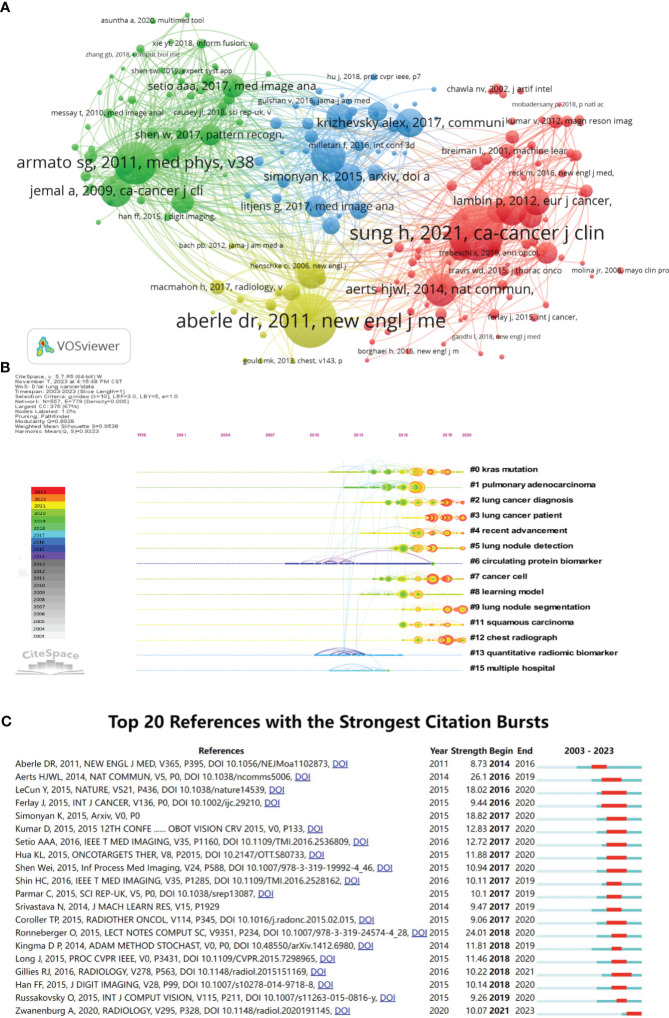
Analysis of reference citations (The circle represents the number of citations. The line represents two articles cited by the same article.). **(A)** Co-citation analysis of references (The colors represent the clustering of references.). **(B)** Timeline diagram of references (The color represents the average time the reference was cited.). **(C)** Top 20 references cited in burst.

All the articles collectively cite 91,880 references, with 201 references cited at least 30 times. We imported the references cited more than 30 times into VOSviewer for co-citation analysis and visualization (**Figure 8A**). The focus is divided into four main clusters: articles in the red and blue clusters are mainly related to computers and artificial intelligence, with references specifically providing technical support and methodological considerations, where the red cluster primarily focuses on imaging genomics, and the blue cluster primarily focuses on deep learning and neural networks. The yellow and green clusters emphasize the application of AI in LC, particularly in lung nodule detection and early lung cancer screening. **Table 6** contains the top 10 most cited references. The most cited is Hyuna Sung et al., with 342 citations, focusing on epidemiological data on cancer. Next are Denise R. Aberle et al. and Samuel G. Armato 3rd et al., with 334 and 265 citations, respectively. These 10 references can be categorized into three types: epidemiology of cancer, methodological studies on AI, and studies on the clinical application of AI in lung cancer. We can depict the categorization and temporal distribution of references through a timeline chart (**Figure 8B**), revealing a predominant surge in publications post-2013. Over the last five years, a substantial number of highly cited articles have surfaced, significantly influencing the integration of artificial intelligence in the realm of lung cancer research. As delineated by clustering outcomes, the cited literature can be delineated into 15 distinct categories, predominantly emphasizing the selection of research subjects and methodologies. The primary research focus is lung cancer, further stratified into specific subtypes, namely lung adenocarcinoma (Cluster 1), lung squamous cell carcinoma (Cluster 11), and lung nodules (Cluster 5/9). Outcome indicators are oriented towards the diagnosis and differential diagnosis of lung cancer (Cluster 2), encompassing histological classification, gene phenotypes, and mutations (Cluster 0), as well as treatment prognosis. The array of research methodologies exhibits diversification and can be granulated into specific domains such as radiomics (Cluster 12), metabolomics (Cluster 6), pathomics, machine learning, and deep learning (Cluster 8), alongside collaborative endeavors involving multiple healthcare institutions (Cluster 15). This clustering underscores the comprehensive exploration of lung cancer research facets, spanning diagnostic modalities, classification approaches, and prognostic assessments, facilitated by a spectrum of advanced research methodologies.

**Table 6:** The top ten articles with the most total citations.

Rank	Paper	Journal	First Author	Year	Total Citations	TC per Year
1	Classification and mutation prediction from non-small cell lung cancer histopathology images using deep learning	NAT MED	COUDRAY N	2018	1196	299.00
2	Multiplatform analysis of 12 cancer types reveals molecular classification within and across tissues of origin	CELL	HOADLEY KA	2014	964	120.50
3	End-to-end lung cancer screening with three-dimensional deep learning on low-dose chest computed tomography	NAT MED	ARDILA D	2019	799	266.33
4	Pulmonary Nodule Detection in CT Images: False Positive Reduction Using Multi-View Convolutional Networks	IEEE T MED IMAGING	SETIO AAA	2016	738	123.00
5	Artificial intelligence in cancer imaging: Clinical challenges and applications	CA-CANCER J CLIN	BI WL	2019	664	221.33
6	Machine Learning methods for Quantitative Radiomic Biomarkers	SCI REP-UK	PARMAR C	2015	610	87.14
7	Deep learning for chest radiograph diagnosis: A retrospective comparison of the CheXNeXt algorithm to practicing radiologists	PLOS MED	RAJPURKAR P	2018	528	132.00
8	Predicting non-small cell lung cancer prognosis by fully automated microscopic pathology image features	NAT COMMUN	YU KH	2016	526	87.67
9	Radiomics in medical imaging-”how-to” guide and critical reflection	INSIGHTS IMAGING	VAN TIMMEREN JE	2020	423	211.50
10	Computer analysis of computed tomography scans of the lung: a survey	IEEE T MED IMAGING	SLUIMER I	2006	380	23.75

**Figure 8C** displays the references that experienced a burst in citations, and it’s evident that there is a spike in references experiencing a burst in citations after 2017, indicating rapid development in the field of AI in LC after 2017. The reference with the highest burst intensity is Aerts et al (25). who, in 2014, demonstrated through radio genomic analysis that prognostic radiomic features capturing intra-tumor heterogeneity are related to underlying gene expression patterns.

## 4 Discussion

With the advancement of hard drives and semiconductors, the capabilities of big data storage and dataset-based computer modeling have become more potent, paving the way for the goal of computers that can simulate human interaction (4). In tandem, AI technology has emerged and rapidly evolved, finding applications across various disciplines. The field of lung cancer, being one of the world’s challenges, is no exception. Bibliometrics allows for the analysis of authors, institutions, countries, and references in the WOSCC bibliographic database, providing insights into a specific research area and visualizing it through tools like Citespace and VOSviewer. This research methodology offers a more comprehensive analysis of literature and presents more visual results than a typical systematic review. In the realm of AI in lung cancer, this study employs bibliometrics as its initial approach to explore the applications and developments in the field over the past two decades and to speculate on future research trends.

In the initial stages, the development of AI in lung cancer research was slow, with an annual publication output of fewer than 30 papers before 2015. After an exploratory period from 2015 to 2017, there was a steady increase, and the growth rate exceeded 100 papers per year. It is projected that by 2023, the annual publication output in this field will surpass 1000 papers (**Figure 2**). This phenomenon indicates that the field is currently experiencing rapid development. The year 2017 marks a crucial turning point in the development of AI in LC, representing a culmination of earlier accumulations and aligning with the trends of the era. On one hand, the emergence of DL and convolutional neural networks (CNN) has led to breakthroughs in processing techniques, especially for complex data such as medical images (26). Simultaneously, the establishment of large-scale public databases related to LC, such as The Cancer Genome Atlas (TCGA), The Cancer Imaging Archive (TCIA), and the LIDC/IDRI (11) Lung Nodule CT Image Database, has facilitated the prominence of genomics and radiomics. On the other hand, in 2017, the victory of AlphaGo over world Go champion Lee Sedol garnered global attention and admiration. Major economic powers worldwide accelerated their deployment of artificial intelligence, propelling the climax of AI development to new heights (27). The attention to AI research in the LC field also increased, with an annual average growth rate of 70.39%, particularly evident in 2020 (**Figure 2**). Although the publication output for 2023 is not fully accounted for due to the study’s timeline, based on the current trends, the application of AI in LC is expected to remain a future research hotspot and will continue to receive attention.

The top two countries in terms of publication output in this field are China and the United States, aligning with the national rankings for lung cancer incidence (1). These two countries contribute to over 60% of the total global publication output, revealing significant research disparities in this field among countries worldwide. China and the United States hold a decisive advantage compared to other nations. Chinese scholars have a total publication output of 1173 papers, but the average citation frequency per paper is relatively low at 15.7 times per paper, similar to other Asian countries such as South Korea and India. However, there is still a gap compared to European and American countries, indicating that the quality of Chinese papers needs improvement compared to their Western counterparts. Through in-depth analysis of the data, it was found that China’s annual publication output began to grow rapidly only in 2017 and surpassed the United States after 2019, reaching twice that of the United States by 2022. This suggests that China started later in this field but has developed rapidly, which may be one of the reasons for the lower average citation frequency. The United States ranks second in publication output but first in total citations, indicating its central position in this field. However, publication output and citation rates are just indicators of research impact, and careful interpretation should consider other factors such as the primary research language, international collaboration, academic achievement policies, language bias in indicators, and publication bias of journals.

**Figures 3C, D** illustrate cooperation between different countries. Among the top 10 countries in this field, most research from countries other than the Netherlands is conducted as single-country studies, indicating a need for stronger international collaboration in AI in lung cancer. There is extensive collaboration between China and the United States, radiating to the North America, East Asia, and Australia regions, indicating that China and the United States, as leading countries in this field, have significant international appeal and influence. Close collaboration is observed among European countries like the Netherlands, Germany, England, and Italy, while strong collaboration exists between Asian countries like India and South Korea. This suggests that collaboration tends to be regional, but there is less collaboration between regions, likely influenced by language and geographical factors.

Among the top 10 institutions, China has 7, while the United States, the Netherlands, and South Korea each have 1. China boasts numerous research institutions and publications, largely due to the robust support for artificial intelligence applications from the Chinese government in recent years. This support spans across various fields involving artificial intelligence (28–31), indicating that China’s research in this field may lead in the future with continuous investment. However, it’s noteworthy that Maastricht University and Harvard Medical School have significantly higher average citation counts of 68.07 and 66.94, respectively, far surpassing other institutions in the top ten. This suggests that they are central institutions in this field. This distinction is mainly attributed to the contributions of Professor Philippe Lambin’s team at Maastricht University and Professor Hugo J.W.L.Aerts’s team at Harvard Medical School. They jointly published the groundbreaking paper “Radiomics: extracting more information from medical images using advanced feature analysis” in 2012, introducing the concept of “radiomics” and ushering in a new era for the application of artificial intelligence in medical imaging (12). Subsequently, they successively published a series of studies (32–35) applying machine learning and deep learning to radiomics in lung cancer, which garnered widespread sharing and citation.

Generally, authors with higher citation counts are considered to have a greater impact than those with lower citation counts, and authors who are co-cited may be concentrated in related research areas. Looking at author contributions and co-cited authors (**Table 4**), the most prolific author in this field is Qian W, with 20 publications, primarily focused on Cancer Computer-Aided Diagnosis (CAD) systems. When considering co-cited authors, Armato SG is the most frequently cited and central co-cited author, indicating his significant role in this field. It’s noteworthy that more than half of the active top 10 authors come from the Asian region (4 from China, 2 from South Korea), but all of the top 10 co-cited authors are from Western countries. Furthermore, in the comprehensive analysis of institution and author collaboration networks (**Figures 4C** and **5C**), we observe that collaboration among authors in the Asian region is mostly limited to the same country. Even on a global scale, only a few highly influential institutions and authors engage in international collaboration. This suggests that research in the field of AI in lung cancer is still relatively dispersed, lacking international exchange. International collaboration and author influence are complementary, and authors such as Philippe Lambin, Hugo J.W.L.Aerts, Issam EL Naqa, and Weiming Li are successful figures in the field, significantly influencing other authors. Their teams could be excellent potential collaborators for researchers.

Publications are the carriers of research achievements, and effective scientific communication requires publishing research results in internationally peer-reviewed journals. Therefore, through the analysis of the distribution of journal sources, researchers can quickly identify the journals most suitable for their papers (36). The analysis of journals and co-cited journals (**Table 7**) reveals that, except for *the International Journal of Imaging Systems and Technology*, the top 20 journals are all excellent journals with a JAR ranking of Q2 and above. Among them, the top 3 journals in terms of publication volume are *Frontiers in Oncology*, *Scientific Reports* and *Cancers* all with over 80 publications, significantly higher than other journals. This indicates that these journals prioritize research in this field, and scholars in the field can give priority to publishing their findings in these journals. In the field of Radiology and Imaging, the top journal is *Radiology* with the highest co-citation frequency. Additionally, other highly influential medical journals such as *New England Journal of Medicine*, *Nature* and *CA-A Cancer Journal for Clinicians* are also listed. This, to some extent, reflects that the application of artificial intelligence is an important research direction in the field of lung cancer. Regarding the distribution across disciplines, in addition to medical-related journals, there are also journals in the fields of computer science and engineering. This indicates that the application of AI in LC is an interdisciplinary field that requires collaboration across multiple disciplines.

**Table 7:** The top 10 references with the most citations.

Rank	Paper	Journal	First Author	Year	Total Citations
1	Global Cancer Statistics 2020: GLOBOCAN Estimates of Incidence and Mortality Worldwide for 36 Cancers in 185 Countries	CA-CANCER J CLIN	SUNG H	2021	342
2	Reduced lung-cancer mortality with low-dose computed tomographic screening	NEW ENGL J MED	ABERLE DR	2011	334
3	The Lung Image Database Consortium (LIDC) and Image Database Resource Initiative (IDRI): a completed reference database of lung nodules on CT scans	MED PHYS	ARMATO SG	2011	265
4	U-Net: Convolutional Networks for Biomedical Image Segmentation	LECT NOTES COMPUT SC	RONNEBERGER O	2015	212
5	Decoding tumour phenotype by non-invasive imaging using a quantitative radiomics approach	NAT COMMUN	AERTS HJWL	2014	201
6	Cancer statistics, 2009	CA-CANCER J CLIN	JEMAL A	2009	196
7	Radiomics: Images Are More than Pictures, They Are Data	RADIOLOGY	GILLIES RJ	2016	185
8	ImageNet Classification with Deep Convolutional Neural Networks	COMMUNICATIONS OF THE ACM	KRIZHEVSKY A	2017	180
9	End-to-end lung cancer screening with three-dimensional deep learning on low-dose chest computed tomography	NAT MED	ARDILA D	2019	173
10	Very deep convolutional networks for large- scale image recognition	ARXIV	SIMONYAN K	2015	170

The analysis of keywords provides another perspective on the development process and trends in the field. To gain a macroscopic understanding of the research hotspots and frontiers of AI in LC, we conducted a visual analysis of high-frequency keywords (**Figure 6**). Representative keywords include “lung cancer,” “classification,” “cancer,” “machine learning,” “deep learning,” and “diagnosis,” indicating that these topics are the research hotspots in this field. Currently, AI applications in LC mainly focus on identification, diagnosis, and therapeutic prediction, with machine learning and deep learning being the most commonly used methods.

Further keyword clustering analysis using CiteSpace was conducted. Noun terms were extracted from the titles of cited literature as labels for clustering, employing the Log-Likelihood Ratio (LLR) algorithm as the extraction method. As shown in **Figure 7A**, the keyword cluster view indicates that “cell lung cancer” #0 and “deep learning” #1 are the largest clusters, suggesting that the application of deep learning in non-small cell lung cancer may be a mature and significant topic in this research field. Currently, deep learning has been widely applied in the clinical diagnosis (16, 37, 38), treatment (35, 39) and prognosis prediction (34, 40) of lung cancer. Simultaneously, the Timeline View analysis (**Figure 7B**) reveals that AI in the field of lung cancer has consistently focused on clinical applications. As detection devices advance and treatment methods evolve, research priorities gradually shift towards early screening, immunotherapy, risk prediction, and other areas. After undergoing early theoretical research and technological exploration, AI has been widely applied in various fields related to lung cancer, including CT imaging, pathological images, genomics, etc., achieving encouraging results and providing accurate guidance and support for clinical early diagnosis and treatment decisions. The duration of keyword prominence was longer before 2016, but became shorter after 2016 (**Figure 7C**). This phenomenon indicates a slow development of AI in LC before 2016, followed by a rapid development phase after 2016, attributed to accelerated technological iterations leading to shorter bursts of prominence. Additionally, emerging terms post-2020, such as shape, volume, radio genomics, predominantly focus on the analysis of imaging data. This suggests that CT and PET/CT, as the most crucial non-invasive diagnostic tools for lung cancer, still hold significant research value. Imaging genomics and related multi-omics studies are identified as the forefront direction in AI research for lung cancer.

Overall, the application of AI in LC can be broadly categorized into three directions. The first category involves the application in early screening and identification of lung cancer. Detection of pulmonary nodules is crucial in low-dose CT screening for lung cancer, and efficient detection significantly enhances the risk assessment of lung cancer. Jiang et al. (41) designed a four-channel convolutional neural network model based on multiple sets of patches cut from lung CT images, which effectively detects pulmonary nodules. Compared to solid solitary nodules, ground-glass opacity (GGO) nodules are more likely to be malignant. He et al. (42) used a 3D CNN to detect the position of GGO nodules and classify lesions (benign or malignant), achieving a competition performance metric (CPM) of 0.817. A portion of metabolic by-products released by human tissues enters the blood, undergoes substance exchange in the lungs, and is expelled from the body through the respiratory tract. Therefore, exhaled breath to some extent can reflect the body’s disease status. The relationship between volatile organic compounds (VOCs) in exhaled breath and lung cancer is a focus of research (43, 44). Researchers defined an instrument, known as an electronic nose (45), composed of an array of electronically sensitive sensors and a pattern recognition system capable of identifying simple or complex odors. In the study (46), the electronic nose was used to distinguish between lung cancer patients and healthy individuals, achieving a discrimination sensitivity of 81% and specificity of 91%. The highest sensitivity, reaching 92%, was observed in stage I lung cancer.

The second category of application involves the use of AI in lung cancer classification. Son et al. (47) found that radiomic features contribute to differentiating invasive adenocarcinoma from *in situ* and minimally invasive adenocarcinoma. Wu et al. (33) employed machine learning methods to explore the predictive performance of radiomic features for lung cancer histological subtyping (adenocarcinoma and squamous cell carcinoma). The results showed that 53 radiomic features were significantly correlated with lung cancer histological subtypes, indicating substantial potential for radiomic features in predicting lung cancer histological subtypes.

The third category involves the application of AI in the prognosis of lung cancer treatment. Deng et al. (48) developed a model based on deep learning and pre-treatment CT for a multicenter prognostic study to predict the survival benefits of epidermal growth factor receptor tyrosine kinase inhibitors (EGFR-TKI) and immune checkpoint inhibitors (ICI) in stage IV non-small cell lung cancer (NSCLC) patients. The model increased the diagnostic accuracy of clinicians with two years of experience from 47.91% to 66.32% and clinicians with five years of experience from 53.12% to 61.41%. Wang et al. (49) developed a prognosis model based on DL and pathological images of lung adenocarcinoma, achieving favorable predictive performance. Cui et al. (50) integrated multi-omics information into the actuarial deep learning neural network (ADNN) architecture for the joint prediction of radiotherapy outcomes, radiation pneumonitis, and local control in stage III NSCLC patients. The results surpassed traditional normal tissue complication probability/tumor control probability models (C-index = 0.660 vs 0.613/0.569).

These studies demonstrate that machine learning and deep learning are currently the most commonly used methods. With the optimization of algorithms and the progress of multi-center studies involving multiple omics, the predictive capabilities of AI have significantly improved. This enhancement effectively increases the clinical diagnostic efficiency and prognosis accuracy of lung cancer, assisting physicians in making correct clinical decisions.

Based on the above analysis, our bibliometric study systematically analyzed the basic situation, research hotspots, and trends of Artificial Intelligence in the field of Lung Cancer from a visual perspective. Therefore, the results of this bibliometric study are objective and accurate, providing comprehensive guidance for clinical physicians and scholars engaged in research in this field. Given the global expansion of digital networks and the continuous innovation of AI technology, the role and advantages of AI in clinical diagnosis and prognosis prediction for LC are becoming increasingly prominent. The application of AI in the field of LC is undoubtedly a current research hotspot and a major research direction for scholars in the coming years.

However, the application of artificial intelligence technology in the field of lung cancer still faces some limitations and challenges. AI requires large sample sizes to support its applications, with the demand for natural images in public domains often reaching tens or even hundreds of thousands. For instance, the Objectron, an open-source 3D object dataset released by Google AI in 2020, already contains 15,000 short video samples and over 4 million annotated images collected from five continents and ten countries. In contrast, in the medical field where higher precision is required, AI datasets typically consist of fewer than a thousand cases and are often retrospective. While data augmentation methods such as flipping and cropping can be employed to expand the dataset (51), prospective multicenter studies with large samples are crucial to demonstrate the reliability of AI models and their clinical utility in the real world.

The protection of patient privacy information limits data collection and sharing. Methods like the Three-Dimensional Shearlet Intuition Fuzzy Algorithm (STIF) (52) have been introduced to address this issue. Additionally, due to variations in data sources, including differences in race, disease severity, and acquisition device parameters, algorithms may vary significantly (53). Therefore, international consensus is needed to guide methodology, and international multicenter studies with large samples are necessary to validate the accuracy of models.

The end-to-end nature of deep learning obscures the data processing process within the model, leading to a lack of interpretability. Before deploying deep learning-based lung cancer models in clinical practice, legal responsibilities and patient safety issues must be considered. Moreover, the complexity of deep learning algorithms implies high hardware requirements and a need for expertise in computer science. Therefore, fostering communication between computer professionals and medical experts and promoting high-quality collaboration between the field are essential.

## 5 Limitations

There are several noteworthy limitations to our study. Firstly, our reliance solely on the WoSCC database implies the potential omission of relevant papers from other databases (21). However, integrating various databases for analysis poses challenges due to limitations in bibliometric software. We have elucidated our rationale for selecting WoSCC as our primary database in the Methods section. Secondly, the implementation of stringent search strategies and the restriction to English-language literature may result in data loss, introducing research bias and diminishing credibility. Lastly, the dynamic nature of databases, coupled with the timing of our study, raises the possibility of underestimating the impact of recently published high-quality articles due to suboptimal citations (54, 55). Additionally, some literature from 2023 may not have been incorporated into our study. Despite these limitations, our study encompasses the majority of publications on AI applied to LC, providing valuable insights into current research hotspots, evolutionary processes, and trends in this field.

## 6 Conclusions

In conclusion, the widespread application of artificial intelligence in the realm of lung cancer is particularly pronounced in auxiliary diagnosis and prognosis prediction. Research emphasis in this domain is progressively shifting towards non-invasive diagnosis and precision therapy facilitated by deep learning techniques. Notably, China and the United States stand as frontrunners in this field, likely maintaining their leadership positions for the foreseeable future. Nevertheless, there is a need for heightened transnational collaboration, particularly among Asian countries, which should actively cultivate close partnerships with developed nations such as the United States and the Netherlands. Simultaneously, there is a call for enhanced interdisciplinary collaboration between the fields of medicine and computer engineering.

## Data availability statement

The original contributions presented in the study are included in the article/Supplementary Material. Further inquiries can be directed to the corresponding authors.

## Author contributions

RZ: Data curation, Formal Analysis, Writing – original draft, Writing – review & editing. TG: Software, Visualization, Writing – original draft, Writing – review & editing. JL: Data curation, Formal Analysis, Writing – original draft. ZL: Software, Writing – original draft. XT: Writing – review & editing, Funding acquisition, Validation. CZ: Methodology, Visualization, Writing – review & editing. XL: Data curation, Writing – review & editing. YW: Writing – review & editing. LG: Funding acquisition, Writing – review & editing. KH: Funding acquisition, Writing – review & editing.
